# Group-Based Lifestyle Intervention Strategies for Metabolic Syndrome: A Scoping Review and Strategic Framework for Future Research

**DOI:** 10.3390/medicina57111169

**Published:** 2021-10-28

**Authors:** Muhammad Daniel Azlan Mahadzir, Kia Fatt Quek, Amutha Ramadas

**Affiliations:** Jeffrey Cheah School of Medicine and Health Sciences, Monash University Malaysia, Bandar Sunway 47500, Selangor Darul Ehsan, Malaysia; dr.mdamahadzir@gmail.com (M.D.A.M.); quek.kia.fatt@monash.edu (K.F.Q.)

**Keywords:** metabolic syndrome, lifestyle intervention, group-based intervention, peers

## Abstract

*Background and Objectives*: Group-based lifestyle interventions reap social support benefits and have been implemented among individuals with various chronic diseases. However, there is a lack of consolidated evidence on its approaches to prevent or manage metabolic syndrome (MetS). This scoping review aims to assess the group-based lifestyle interventional strategies for MetS and provide a strategic framework for future research in this area. *Materials and Methods*: Scholarly databases (OVID Medline, SCOPUS, PUBMED, PsycINFO, EMBASE, and Cochrane Central Register of Controlled Trials) and reference lists of included publications were systematically searched using appropriate keywords and MeSH terms. Peer-reviewed articles published from the start of indexing to 31 December 2020 focused on individuals with or at risk for MetS were included. *Results*: Thirteen interventions were identified, with seven conducted among adults with MetS and six in the population at risk for MetS. Three study designs were reported—randomised controlled trials (RCTs), pre–post interventions, and quasi-experiments. Most of the interventions were based in the community or community organisations, multifaceted, led by a multidisciplinary healthcare team, and assisted by peer educators. Waist circumference showed the most promising MetS-related improvement, followed by blood pressure. *Conclusions*: There is growing evidence supporting group-based lifestyle interventions to improve MetS-related risk factors. In summary, four strategies are recommended for future research to facilitate group-based interventions in preventing and managing MetS.

## 1. Introduction

Metabolic syndrome (MetS) is a clustering of biochemical and physical conditions associated with an increased risk for developing type 2 diabetes (T2D) and cardiovascular diseases [1]. Excess abdominal fat is most closely associated with metabolic risk factors and is most likely the initiating factor of risk factor clustering in MetS [2]. These risk factors, namely, impaired blood glucose, dyslipidaemia, and raised blood pressure, are symptoms of metabolic chaos inside the body. A high carbohydrate diet, inadequate hydration, a poor sleeping pattern, and overnutrition were described as strong modifiable risk factors for MetS [3]. Hence, MetS became an ideal target of lifestyle-focused interventions.

Comprehensive and sustainable interventions should incorporate a considerable level of health education, intensive self-management skills training, and behavioural-targeted modification in addition to traditional clinic visits [4]. This can be achieved through an interactive environment, such as supportive groups.

Group-based interventions have been documented to manage various metabolic conditions, especially among those with T2D and obesity [5,6]. Although evidence suggests that group interventions have a therapeutic benefit beyond providing patients with information and education, studies on its effectiveness need further exploration [7]. For example, while group-based programs effectively improved clinical, lifestyle, and psychosocial outcomes among patients with T2D [5], the interventions themselves have been poorly documented [8]. The designs and effectiveness of these interventions also tend to vary considerably among the studies [6]. However, interventions underpinned with a theoretical framework, education, and social support tend to be more efficacious [9].

In addition to that, peer support has become a common aspect of group-based interventions. While not all group-based interventions use the peer support framework, it has been shown to have beneficial effects on intervention outcomes. Peer support in lifestyle interventions has provided a space for healthcare providers or trained peer leaders to deliver extensive health education and self-management instruction while also allowing for increased adoption and productivity [10]. Peer support combines the benefits of receiving and providing social support, making it superiorly beneficial for adults with similar chronic diseases who need a lifestyle change [10].

This scoping review aimed to summarise evidence based on group-based lifestyle interventions targeted for MetS and to present a strategic research framework for lifestyle intervention on MetS for future research.

## 2. Materials and Methods

We conducted the scoping review according to standard protocols [11,12,13] and the Preferred Reporting Items for Systematic reviews and Meta-Analyses extension for Scoping Reviews (PRISMA-ScR) [14]. Ethical approval was not sought, as the data were publicly available.

### 2.1. Search Strategy

We conducted a systematic literature search in OVID Medline (Alphen aan den Rijn, Netherlands), SCOPUS (Amsterdam, The Netherlands), PUBMED (Bethesda, MD, USA), PsycINFO (Washington, DC, USA), EMBASE (Amsterdam, The Netherlands), and Cochrane Central Register of Controlled Trials (Hoboken, NJ, USA) from the start of indexing to 31 December 2020. We devised a search strategy using the following keywords/MeSH: ((peer) OR (‘peer group’) OR (‘peer support’) OR (‘self-help group’) OR (‘support group’) OR (‘support program’) OR (‘social support’)) AND (intervention) AND ((‘metabolic syndrome’) OR (‘metabolic syndrome X’) OR (‘syndrome X’) OR (‘insulin resistance syndrome’)).

Table S1 shows the search strategy for OVID Medline. The strategy was adopted for the remaining electronic databases.

### 2.2. Study Selection

We screened the records obtained from database searches using Covidence software [15]. The screening process was conducted in a two-step approach, where the title and abstract of the records were first screened. Irrelevant articles were removed, and subsequently, the full texts of the remaining articles were reviewed based on pre-determined eligibility criteria.

To be eligible, the peer-reviewed articles should be focused on (i) individuals with MetS, with MetS defined according to an established guideline, or (ii) individuals identified to be at risk for MetS. Peer-based intervention must have been designed in a group setting, where a peer or community member led the intervention. To improve the inclusivity of the studies, we also included interventions that reported interaction or interactive sessions between participants in a group setting.

Studies that compared interventions focusing on diet, lifestyle behaviour, and/or physical activity levels with conventional dietary advice or no treatment were eligible. Multifaceted interventions with a combination of diet, lifestyle behaviour, and physical activity were also considered. We included interventions with randomised and non-randomised designs regardless of the location, intervention provider, intervention duration, and sample size. Single-armed studies and studies comparing different modalities of group-based interventions were also considered.

Studies with a focus on supplements, functional foods, or pharmacological drugs were excluded. We also excluded studies that did not report pre- and post-intervention changes in at least one MetS parameter. Any publications that were not published in peer-reviewed scientific journals were also not considered. 

Two authors independently conducted the screening, and a third author resolved any conflict in the agreement between the authors. The bibliographies of the included articles were manually searched for additional relevant articles.

### 2.3. Data Extraction and Synthesis

We extracted the following key information from the studies: study design, recruitment location, intervention target, intervention design, sample size, participant age, and MetS-related outcomes. Subsequently, we reorganised the studies according to the setting, intervention, and outcomes that formed the theme of this review.

## 3. Results

### 3.1. Search Results

**Figure 1:**
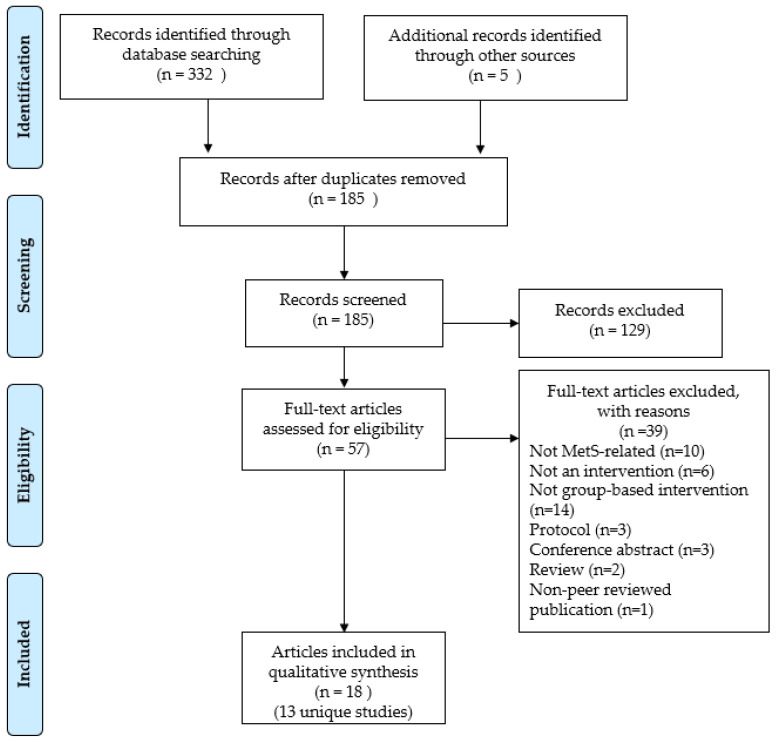
PRISMA flow chart showing the study selection process.

Figure 1 presents the study selection process. The systematic database searches resulted in a total of 332 records, while manual searching resulted in 5 records. After removing 152 duplicates, through the title and abstract screening, 129 irrelevant articles were removed. Subsequently, 57 full texts were available for screening. Finally, 18 articles (representing 13 unique studies) that met the eligibility criteria were included in the review. The included studies are summarised in Tables S2 and S3.

### 3.2. Study Characteristics

Most studies were randomised controlled trials (RCTs) [16,17,18,19,20,21,22,23,24,25], followed by pre–post design [26,27,28,29,30,31,32] (Table 1). Five interventions were conducted in the United States [17,18,26,29,30,31,32]. Seven studies were conducted among adults with MetS or at risk for the syndrome without other medical conditions [16,17,18,24,27,28,30,33]. Three studies were conducted among adults with other medical issues and increased risk for MetS [25,29,31,32], while two interventions were conducted among obese adults [19,20,23]. National Cholesterol Education Program (NCEP) Adult Treatment Panel (ATP) III (NCEP ATP III) criteria were the most commonly used MetS definition for patient inclusion [16,21,22,23,26], followed by International Diabetes Federation (IDF) [17,18,19,20] and Joint Interim Societies (JIS) Harmonised criteria [27,28].

### 3.3. Intervention Characteristics

Most studies recruited their participants at the community level, either by media or the use of flyers [19,20,23,29]; community-based health screenings [21,22,24,28]; or from community-based organisations, such as churches [30,31] (Table 2). The study by Sanee et al. [33] was the only study that recruited participants from a workplace setting, while the study by Bo et al. [16] was the only study that drew its participants from an existing cohort. Seven studies had a multidisciplinary team of health professionals delivering its intervention program [16,17,18,24,29,31,33]. All studies included in this review documented group-level interaction between the study participants during the intervention. Eight interventions utilised the concept of peer educators or community volunteers [19,20,23,25,27,28,29,30,31,32,33]. Most interventions took place in the community [19,20,21,22,23,27,28,29], followed by community or primary care clinics [16,17,18,26,30], and lasted for three months or less [17,18,25,28,30,31,33].

### 3.4. Multifaceted Interventions

Mahadzir and colleagues [27,28] reported the only peer group-based multifaceted intervention among adults with MetS (N = 48). The study was led by a nutritionist and set within a local community in Malaysia. The weekly nutrition and lifestyle intervention modules were developed using the Health Belief Model and community-specific approaches. Three other multifaceted interventions [29,30,31,32] were conducted among adults at risk for MetS and with the involvement of peer leaders or community volunteers. While Gill et al. [31] and Buckley et al. [30] reported short-term interventions, Bazzano and colleagues [29] conducted a seven-month community-based intervention developed using the social cognitive theory in a larger group of at-risk adults with developmental disabilities (N = 431). Buckley et al.’s intervention was conducted among uninsured Hispanic adults with a low-income background (N = 192) with materials that were developed for a target population with low English proficiency [30]. The intervention was delivered by local peer educators who had undergone extensive training in case management, community outreach, and health education.

Similarly, Gill et al.’s intervention among adults with serious mental illness also used extensively trained peer wellness coaches [31,32]. Their intervention used an interactive module with a peer-based coaching session, focusing on health education, personal goal setting, and physical activity to improve readiness to change health behaviours. In this study, stages of change approaches were used to assess the readiness to change behaviours.

### 3.5. Weight Management Interventions

Three studies reported on weight management intervention among adults with MetS, where two studies were reported in the United States [17,18,19,20] and one in Australia [26]. Ma and colleagues [17,18] utilised the Group Lifestyle Balance (GLB)^TM^ program, an adapted 12 sessions of the Diabetes Prevention Program (DPP) lifestyle curriculum developed using social cognitive theory and the Transtheoretical Model of Change. A multidisciplinary team delivered the program to 241 individuals with MetS through 12 weekly highly interactive classes. Pettman et al.’s four-month intervention “Shape Up for Life” was a structured non-prescriptive lifestyle education program for obese adults with MetS (N = 153) [19,20]. The program was adapted from Stanford’s Chronic Disease Self-Management Program (CDSMP) [34], centred on the self-efficacy theory and developed based on the Australian national diet and physical activity guidelines. The intervention was coordinated by a nutritionist and led by peer leaders. Another intervention led by a physician [26] was conducted among a smaller group of patients with MetS (N = 22) for ten weeks using the Cooperative Health Care Clinic (CHCC) module. The CHCC was developed based on the established Lifestyle, Exercise, Attitude, Relationships, and Nutrition (LEARN) program for weight control curriculum [35]. While the sessions were not peer-led, facilitated group discussions and peer learning were encouraged during and after the presentation. Patients were also encouraged to share their experiences and problem-solving strategies.

### 3.6. Nutrition and Physical Activity Interventions

Chang et al. [23] reported the only study focusing on community-based physical activity among metabolically abnormal obese adults with MetS (N = 131), led by a multidisciplinary team and community volunteers. This six-month Taiwanese intervention included providing exercise environments and skills and reminders from the volunteers. The only study focusing on nutrition education for older adults with MetS (N = 47) was reported in Malaysia [21,22]. The community-based intervention was dietitian led and provided nutrition education via group counselling sessions, talks, and cooking and exercise demonstrations using a specifically developed healthy ageing package.

Few studies reported lifestyle intervention with a combined focus on nutrition and physical activity in MetS [16,24,25,33]. The only such intervention among the MetS population (N = 335) was conducted in Italy for 12 months [16]. The lifestyle intervention was conducted in a primary or community clinic setting with a team-based approach and interactive group sessions. The remaining nutrition/physical activity interventions were conducted among the at-risk population. Yamashiro et al.’s intervention was conducted in a health promotion centre (N = 137), led by a multidisciplinary team [24]. While the study did not engage peer leaders, the intervention did include group discussion sessions. Sanee et al.’s workplace intervention among Thai women (N = 100) was conducted in weekly peer-led individual support discussions and monthly meetings for three months [33]. A more recent Kenyan study among type 2 diabetes patients at risk for MetS (N = 143) focused on nutrition and physical activity [25]. The two-month intervention was led by a nutritionist and peer educators. Nutrition education, including diabetes-related nutrition, food portion control, and healthier food choices, and individualised meal planning were provided, in addition to peer-to-peer support. A lesson on physical activity was given at the end of the program for patients to accumulate at least 150 min of moderate-intensity exercise a week.

### 3.7. Study Outcomes

**Figure 2:**
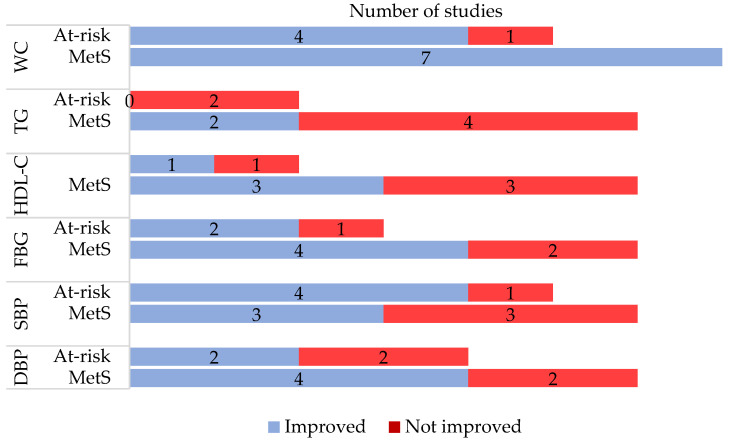
Metabolic syndrome-related outcomes as reported in the included studies. MetS = metabolic syndrome; WC = waist circumference; TG = triglyceride; HDL-C = high-density lipoprotein cholesterol; FBG = fasting blood glucose; SBP = systolic blood pressure; DBP = diastolic blood pressure.

Table S4 presents all study outcomes reported by the studies, while Figure 2 summarises MetS-related outcomes reported in the included studies. Almost all studies that reported on waist circumference found a significant reduction post-intervention. This is followed by improved systolic blood pressure, as shown in three MetS and four at-risk studies, and diastolic blood pressure (four MetS and two at-risk studies). The most negligible improvement was demonstrated in triglyceride levels, with only two MetS studies showing significant improvement.

## 4. Discussion

Non-communicable diseases (NCDs) disproportionately affect people in low- and middle-income countries. More than 85% of global NCD deaths occur in low-resource settings [36]. The exorbitant costs of NCDs, including prolonged, expensive treatment, pose a significant economic burden worldwide, especially in developing countries [37]. Therefore, cost-effective innovations to manage and prevent NCDs, including MetS, are required. This review highlighted 13 studies of variable designs exploring group-based lifestyle interventions in MetS. Most of the studies employed existing diabetes self-management programs and published national guidelines and were primarily focused on clinical endpoints.

Despite the need to scale up lifestyle-based interventions for NCDs, very few interventions met the benchmark [38]. The inclusion of an appropriate behavioural theory in intervention development among MetS patients was beneficial [39]. Based on our review, several psychological theories have been used in program development, including the Social Cognitive Theory, Transtheoretical Model of Change, the Health Belief Model, and self-efficacy theory. Hence, it can be concluded that there is yet to be conclusive evidence to point out the most suitable theory or model for the clustering of risk factors or MetS as a whole [40]. The selection of behavioural theory in an intervention should be made based on the objective of the intervention and the factor to be intervened.

**Figure 3:**
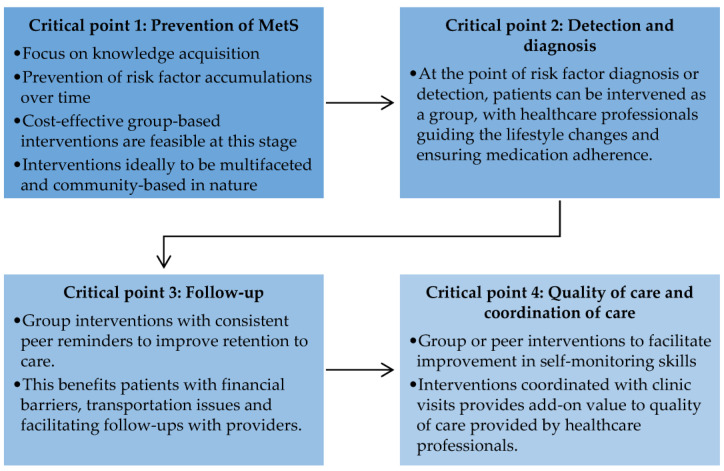
Critical points for future research in the prevention and management of metabolic syndrome.

There is a need for a systematic evaluation of behavioural approaches in lifestyle intervention to help researchers learn about fundamental elements that may improve or halt behavioural change among adults with MetS [41]. This understanding can assist researchers in designing a better framework to intervene in different populations throughout the world. As the information and knowledge of peer-based intervention on NCDs, particularly MetS, is still in infancy, research is needed on all dimensions, including designing, implementing, and evaluating different peer support models to meet the needs of diverse populations in various settings [40,42]. This review suggests a set of research directions for the prevention and management of MetS (Figure 3).

### 4.1. Critical Point 1: Prevention of Metabolic Syndrome

Poor knowledge of preventive measures is a crucial contributor to the rising incidence of MetS. MetS itself is a non-consensus disease, as it is perceived as a clustering of metabolic risk factors [43]. Hence, the effort to prevent MetS is overshadowed by intervention involving pre-diabetes and overweight and obese adults, albeit related [43]. However, the early prevention of MetS could reduce the accumulating effect of more risk factors, which may become harder to manage with time [41,44]. For this purpose, future studies could adopt group-based interactive interventional designs, such as those involving peer support, as these designs are cost effective and feasible [44].

### 4.2. Critical Point 2: Detection and Diagnosis

Although trained health professionals as peer leaders have been leveraged to address the risk factors for MetS in developed countries, this was shown to be inefficient [45,46]. Group-based intervention may increase awareness through education and behaviour change through inter-peer communication. Such systems can be tailored to provide information regarding MetS frequently to remind all about healthy lifestyle practices and medication adherence under the supervision and guidance of healthcare professionals. This has been demonstrated in interventions with decision support for hypertension [47] and T2D [48] in developing countries.

### 4.3. Critical Point 3: Follow-Up

Group-based intervention improves retention to care, as inter-peer communications often deal with consistent reminders about lifestyle behaviour change, daily monitoring, medication, and upcoming interactive sessions [40]. Peer reminders, for example, can play a role in promoting sustained lifestyle modification among adults with MetS. From a broader perspective, peer-based intervention can also support retention to care by assisting patients with financial barriers and transportation issues and by facilitating follow-ups with providers [49]. Research on prospective peer support intervention following clinic visits should elucidate the efficacy of peer support in promoting sustained lifestyle changes.

### 4.4. Critical Point 4: Quality of Care and Coordination of Care

The peer-based program can support patient education, training, work planning, decision support, and treatment adherence in addition to routine clinic visits [10,50]. This added value to the quality of care provided by healthcare professionals. Peer-based programs, such as weekend classes, allow peer leaders to inform peers on MetS and improve self-monitoring skills following official clinic visits [51]. While the evidence of peer-based intervention points out improvement in MetS control, the outcomes of coordinated care between healthcare professionals and peers are still unknown. The details of coordinated care provided by peer-based intervention in addition to clinic visits are essential to ensure the continuity of care and informed decision making in MetS.

Our review found that triglycerides and high-density lipoprotein cholesterol (HDL-C) showed the most negligible improvements. This is not surprising since these lipid alterations are accompanied by the predominance of small yet dense low-density lipoprotein cholesterol (LDL-C) and are usually under recognised in MetS. These three lipid alterations constitute the so-called lipid triad or atherogenic lipoprotein phenotype, which is the main feature of MetS [52]. Small LDL-C particles are closely associated with atherosclerosis formation and progression [53] and are strong predictors of future cardiovascular and cerebrovascular diseases in individuals with MetS [54]. Therefore, early recognition of these lipid alterations in MetS can contribute to proper management and treatment to reduce cardiometabolic risk [55].

Strategic future studies are crucial to elucidate the optimal interventional strategies for MetS to be aligned with the target set by clinical standards. The urgency for high-quality evidence stems from the need to inform many important decisions regarding the diagnosis, prevention, and treatment of MetS, which facilitates peer support alongside clinical practice, highlighting the need to incorporate implementation research, monitoring, and evaluation in peer-based research. It may assist stakeholders and policymakers in evaluating innovations and strategies that merit incorporation into the existing health system and adding further investment.

### 4.5. Study Limitations

This review has several limitations. Articles are limited to English language publications due to a lack of qualified translators in other languages. As this study was conducted to elucidate the interventional strategies and formulate a framework for future studies in these aspects, we chose to conduct a scoping review instead of a systematic review. A systematic review accompanied by a meta-analysis will be appropriate to highlight the outcome benefits of group-based interventions, and this would be a focus for another study.

## 5. Conclusions

This scoping review gathered 13 studies on group-based lifestyle interventions among adults with MetS and the at-risk population. There was considerable heterogeneity in the intervention designs, and the role of the peer leaders varied considerably. Waist circumference showed the most significant improvement post-intervention, while evidence on intervention effectiveness on moderating factors such as nutrition and lifestyle changes is scarce. Hence, we provided suggestions for framing future research at different critical points in the prevention and management of MetS, including the training of peer leaders and local stakeholders to integrate peer support as a complement to standard care. Since MetS is a chronic lifestyle-related disease that incurs an economic burden to the healthcare sector, a practical, cost-effective public health approach is needed to overcome the rising prevalence.

## Figures and Tables

**Table 1:** General characteristics of the included studies (N = 13).

		Population			References ^a^
		All (N = 13)	Metabolic Syndrome (*n* = 7)	At Risk (*n* = 6)	
Study design	Randomised controlled trials	7	5	2	[16,17,18,19,20,21,22,23,24,25]
	Pre–post	5	2	3	[26,27,28,29,30,31,32]
	Quasi-experiment	1	0	1	[33]
Location	United States	5	2	3	[17,18,26,29,32]
	Malaysia	2	2	0	[21,22,27,28]
	Italy	1	1	0	[16]
	Australia	1	1	0	[19,20]
	Taiwan	1	1	0	[23]
	Japan	1	0	1	[24]
	Thailand	1	0	1	[3]
	Kenya	1	0	1	[25]
Population	Adults	7	4	3	[16,18,24,26,28,30,33]
	Adults with other health issues	3	0	3	[25,29,31,32]
	Obese adults	2	2	0	[19,20,23]
	Older adults	1	1	0	[21,22]
Metabolic Syndrome Criteria	NCEP ATP III	4	4	n/a	[16,21,23,26]
	IDF	2	2	n/a	[17,20]
	Harmonised	1	1	n/a	[27,28]

n/a = not applicable; ^a^ the count may not be reflected by the number of references, as each study can be represented by more than one article.

**Table 2:** Intervention characteristics of the included studies (N = 13).

		Population			References ^a^
		All (N = 13)	Metabolic Syndrome (*n* = 7)	At Risk (*n* = 6)	
Recruitment	Media/flyers	3	2	1	[19,20,23,29]
	Community clinic/primary care	3	2	1	[17,18,25,26]
	Community-based organisation	2	0	2	[30,32]
	Community screening	3	2	1	[21,22,24,27,28]
	Workplace	1	0	1	[33]
	Existing cohort	1	1	0	[16]
Intervention provider	Dietitian/nutritionist/professional with nutrition background	4	3	1	[19,22,25,27,28]
	Physician/medical doctor	2	1	1	[26,30]
	Multidisciplinary team	7	3	4	[18,20,23,24,29,31,33]
Peer educators or community volunteers		8	3	5	[19,20,23,25,27,33]
Intervention setting	Community	5	4	1	[19,20,21,22,23,27,28,29]
	Primary/community clinic	5	3	2	[16,17,18,25,26,30]
	Community-based organisation	2		2	[30,31,32]
	Health promotion centre	1		1	[24]
	Workplace	1		1	[33]
Study duration (months)	3 or less	7	3	4	[17,18,25,26,27,28,30,31,32,33]
	4–5	1	1		[19,20]
	6 or more	5	3	2	[16,21,22,23,24,29]
Intervention focus	Multifaceted	4	1	3	[27,28,29,30,31,32]
	Nutrition and physical activity	4	1	3	[16,24,25,33]
	Weight management	3	3		[17,18,19,20,26]
	Nutrition	1	1		[21,22]
	Physical activity	1	1		[23]

^a^ The count may not be reflected by the number of references, as each study can be represented by more than one article.

## Data Availability

Data sharing not applicable.

## Supplementary Materials

The following are available online at https://www.mdpi.com/article/10.3390/medicina57111169/s1, Table S1: Sample search strategy in OVID Medline, Table S2: Summary of studies conducted among population with metabolic syndrome (*n* = 7), Table S3: Summary of studies conducted among population at risk for metabolic syndrome (*n* = 6), Table S4. Outcomes reported by the included studies (N = 13).
