# Supplementary material for: Group-Based Lifestyle Intervention Strategies for Metabolic Syndrome: A Scoping Review and Strategic Framework for Future Research
Source: Medicina (Kaunas). 2021 Oct 28;57(11):1169. doi: 10.3390/medicina57111169 (PMC8624746; doi:10.3390/medicina57111169)
Supplement: Supplementary file 1 [file medicina-57-01169-s001.zip › Table S1.pdf]

Table S1. Sample search strategy in OVID Medline.

#	Searches	Results
1	exp Peer Group/ or peer support.mp. or exp Social Support/	93982
2	peer.mp.	77469
3	support group.mp. or exp Self-Help Groups/	12310
4	support program.mp.	1387
5	1 or 2 or 3 or 4	156163
6	intervention.mp.	548469
7	metabolic syndrome.mp. or exp Metabolic Syndrome/	50376
8	syndrome x.mp.	1885
9	metabolic syndrome x.mp.	340
10	insulin resistance syndrome.mp.	1591
11	7 or 8 or 9 or 10	52839
12	5 and 6 and 11	40
13	limit 12 to yr="1902-2020"	40
