# Supplementary material for: Group-Based Lifestyle Intervention Strategies for Metabolic Syndrome: A Scoping Review and Strategic Framework for Future Research
Source: Medicina (Kaunas). 2021 Oct 28;57(11):1169. doi: 10.3390/medicina57111169 (PMC8624746; doi:10.3390/medicina57111169)
Supplement: Supplementary file 1 [file medicina-57-01169-s001.zip › Table S2.pdf]

Table S2. Summary of the studies conducted among the population with metabolic syndrome (*n* = 7).

	Study / Country / Design	Participants	Age (years)	MetS criteria	Intervention provider / Setting	Study duration (months)	Intervention	Main findings
**1**	Bo et al. [16]<br><br>Italy<br>RCT	335 adults<br><br>*169 intervention*<br>*166 control*	Intervention: M=55.7 (SD=5.7)<br><br>Control: M=55.7 (SD=5.6)	NCEP ATP III	Multidisciplinary team<br><br>Primary / community clinic	12	Lifestyle intervention (diet and physical activity) program (5 sessions) with team-based approach and interactive group sessions.<br><br>*Control: Standard counselling*	Significant reduction in prevalence of MetS, central obesity, hypertriglyceridemia and incidence of diabetes in intervention group at 12 months.
**2**	Ma et al. [17,18]<br><br>United States<br>RCT	241 adults<br><br>*79 coach-led*<br>*81 DVD*<br>*81 control*	M=52.9 (SD=10.6)	IDF	Multidisciplinary team (Registered dietitian, fitness instructor, lifestyle coach)<br><br>Primary care clinic	3	An adapted, 12-session DPP lifestyle intervention curriculum, Group Lifestyle Balance (GLB)™. The program was delivered face-to-face in 12 weekly highly interactive classes to coach-led intervention participants, or via a home-based DVD.<br><br>Theory: Social Cognitive Theory and the Transtheoretical Model of Behaviour Change<br><br>*Control: Usual care*	Greatest reduction in BMI seen in coach-led group. 35.9% participants in coach-led group achieved the 7% DPP-based weight loss. Coach-led and DVD groups achieved greater net improvement in WC and FBG.
**3**	Pettman et al. [19,20]<br><br>Australia<br>RCT	153 obese adults<br><br>*103 intervention*<br>*50 control*	M=45	IDF	Study coordinator with nutrition background, peer leader<br><br>Community	4	"Shape Up for Life" - structured but non-prescriptive lifestyle education program that was based on the Australian national diet and physical activity guidelines, developed based on Stanford's Chronic Disease Self-Management Program (CDSMP).<br><br>Theory: Self-efficacy theory<br><br>*Control: Usual care*	Greater improvements in body composition (weight, BMI, body fat mass & percentage, abdominal fat mass, WC), blood pressure (SBP, DBP, mean arterial BP), total cholesterol, LDL-C, physical work capacity, handgrip strength and dietary glycaemic index in intervention group.<br><br>Program recorded high retention rate.
**4**	Greer et al. [26]<br><br>United States<br>Pre-post	22 uninsured adult patients	Osceola Clinic group: M=57.3<br><br>Sumter	NCEP ATP III	Physician<br><br>Community clinics	10 weeks	Cooperative Health Care Clinics (CHCC) module (Lifestyle, Exercise, Attitude, Relationships, and Nutrition (LEARN) Program for Weight Control curriculum) - a weight management program utilizing a therapeutic lifestyle	Significant improvement in WC and knowledge base.<br><br>High patient satisfaction score.

			County group: M=54.2				change approach, addressing nutrition, physical activity, lifestyle changes, goal setting, recording, and helpful devices to achieve goals in an interactive format.<br>Facilitated group discussions and peer learning were encouraged during and after the presentation. Patients were encouraged to share their experiences and problem-solving strategies.	
**5**	Shahar et al. [21,22]<br><br>Malaysia<br>RCT	47 older adults of Malay ethnicity<br><br>*24 intervention*<br>*23 control*	M=66.5 (SD=4.3)	NCEP ATP III	Dietitian<br><br>Community	6	Nutrition education via group counselling sessions, talks, and cooking and exercise demonstrations using a specifically developed healthy aging package.<br><br>*Control: Standard care*	Improvements in WC in older women and total cholesterol levels in older men were seen in the intervention group.
**6**	Chang et al. [23]<br><br>Taiwan<br>RCT	131 metabolically abnormal obese individuals<br><br>*65 intervention*<br>*66 control*	M=55.2 (SD=14.2)	NCEP ATP III	Multidisciplinary team (Nurse, exercise experts), community volunteers<br><br>Community	6	Community-based physical activity program including providing of exercise environments, exercise skills and volunteers' reminding.<br><br>*Control: Provided environment and skills.*	Greater improvement in HDL-C, body weight, BMI, WC, BP and FBG in intervention group.
**7**	Mahadzir et al. [28,29]<br><br>Malaysia<br>Pre-post	48 adults	Md=46 (IQR=11)	Harmonized	Nutritionist, peer educators<br><br>Community	3	An evidence-based and community-specific nutrition and lifestyle behaviour peer support program.<br><br>Theory: Health Belief Model	Overall increases in carbohydrate intake, glycaemic load, physical activity, duration of sleeping, and reductions in total energy and fat.<br><br>Marginal improvements in WC, HDL-C, FBG, BMI, TG, SBP.

M=Mean; SD = Standard deviation; Md = median; IQR = interquartile range; MetS = metabolic syndrome; BMI = body mass index; WC = waist circumference; TG = triglyceride; HDL-C = high density lipoprotein cholesterol; FBG = fasting blood glucose; SBP = systolic blood pressure; DBP = diastolic blood pressure
