# Supplementary material for: Group-Based Lifestyle Intervention Strategies for Metabolic Syndrome: A Scoping Review and Strategic Framework for Future Research
Source: Medicina (Kaunas). 2021 Oct 28;57(11):1169. doi: 10.3390/medicina57111169 (PMC8624746; doi:10.3390/medicina57111169)
Supplement: Supplementary file 1 [file medicina-57-01169-s001.zip › Table S3.pdf]

Table S3. Summary of studies conducted among population at risk for metabolic syndrome (*n* = 6).

	Study / Country / Design	Participants	Age (years)	Intervention provider	Study duration (months)	Intervention	Main findings
**1**	Bazzano et al. [29]<br><br>United States<br>Pre-post	431 adults with developmental disabilities	Majority 40-49	Multidisciplinary team, peer mentors<br><br>Community organization, Community	7	Community-based education and exercise program to increase knowledge, skills, and self-efficacy regarding health, nutrition, and fitness.<br><br>Core aspects: (1) client peer mentoring; (2) interactive health education; (3) supervised physical activity; (4) behavioural modification; (5) one-on-one health management education and advocacy; (6) clinical support; and (7) a supportive social network.<br><br>Theory: Social cognitive theory	Significant improvements in body weight, BMI, WC, physical activity (frequency of exercise and duration), nutritional habits, self-efficacy and life satisfaction were observed.
**2**	Yamashiro et al. [24]<br><br>Japan<br>RCT	137 adults	Intervention 1: M=61.6 (SD=0.8)<br><br>Intervention 2: M=61.9 (SD=0.8)<br><br>Control: M= 60.9 (SD=1.1)	Multidisciplinary team<br><br>Health promotion centre	10	Lifestyle intervention for 4 months (Group 1 & 2) of MS, diet, exercise and behaviour modifications, including group discussion sessions to draw on and learn from the experiences of other people in the group and provided mutual encouragement to maintain the recommended lifestyle modifications.<br><br>Participants were advised to reduce their calorie intake, limit their salt intake, consume fibre-rich foods and perform min 1 hour of aerobic exercise three times a week.<br><br>Group 2 were provided with extended intervention on exercise for another 6 months.<br><br>*Control: Usual care + leaflet with brief motivational advice*	Significantly lower number of MetS components in both intervention groups compared to control at 10- and 34-month.<br><br>Significant improvement in BMI, HDL-C, TBG, HbA1c in both intervention groups at 10 months. Effects on BMI and HDL were sustained at 34 months in both intervention groups.
**3**	Buckley et al. [30]<br><br>United States<br>Pre-post	192 Hispanic low-income, uninsured adults	M=49.8	Medical doctor, trained community health-workers (Navegantes)<br><br>Community clinic, churches	2	Adapted based on ‘‘Thumbs Up!’’ metabolic syndrome workbook. Materials on basic nutrition and metabolic syndrome risk factors were specifically developed for low English proficiency populations.<br><br>Delivered by Navegantes (peer educators) who were trained in a 10-week program on case management, community outreach, and health education specific to the curriculum of the program.	Increase in health literacy in almost 90% the participants.<br><br>Reduction in MetS risk factors (FBG, TC, BMI, WC) in 60% of the participants.

**4**	Gill et al. [31,32]<br><br>United States<br>Pre-post	64 adults with serious mental illnesses	M=50	Multidisciplinary team, peer wellness coaches<br><br>Community, Community clinic, University	2	Weekly program aiming on personal goal-setting, health education, peer wellness coaching, and structured physical activity to improve readiness to change health behaviours, goalsetting abilities, and health behaviours. Each session included an interactive educational module, a small group peer wellness coaching section, and a supported physical activity component.<br><br>*Theory: Stages of Change*	Improvements in BP and WC were seen. Participants also improved on measures of strength and flexibility and reported increased readiness to exercise and make dietary changes.
**5**	Sanee et al. [33]<br><br>Thailand<br>Quasi-experiment	100 adult women	Intervention: M=47.0 (SD=7.3)<br><br>Control: M=48.5 (SD=7.1)	Multidisciplinary team, peer educators<br><br>Workplace	3	Weekly peer-led individual support discussion sessions that included dietary and physical activity advice.	Greater improvement in SBP, DBP, physical activity, MetS knowledge, MetS perception and stress in the intervention group.<br><br>Non-significant improvement seen on WC, BMI and FFQ score.
**6**	Thuita et al. [25]<br><br>Kenya<br>RCT	143 adults with T2D<br><br>*48 nutrition education + peer support (NEP)*<br>*49 nutrition education (NE)*<br>*46 control*	M=56	Nutritionist, peer educator<br><br>Diabetes care centre	2	Nutrition education including diabetes-related nutrition, food portion control, healthier food choices, individualized meal planning, among others were provided, in addition to peer-to-peer support. Lesson on physical activity was given at the end of the program for patients to accumulate at least 150min of moderate intensity exercise a week.<br><br>*NE group – similar content with intervention, except peer support.*<br>*Control – standard care*	Greatest improvements in food intake patterns, physical activity, anthropometry (weight, BMI, WC, HC, WHR), blood lipids (TC, HDL-C), FBG and HbA1c were seen in NEP group.

M=Mean; SD = Standard deviation; MetS = metabolic syndrome; BMI = body mass index; WC = waist circumference; HC = hip circumference; WHR = waist-hip ratio; TG = triglyceride; TC = total cholesterol; HDL-C = high density lipoprotein cholesterol; FBG = fasting blood glucose; SBP = systolic blood pressure; DBP = diastolic blood pressure
