# Supplementary material for: Group-Based Lifestyle Intervention Strategies for Metabolic Syndrome: A Scoping Review and Strategic Framework for Future Research
Source: Medicina (Kaunas). 2021 Oct 28;57(11):1169. doi: 10.3390/medicina57111169 (PMC8624746; doi:10.3390/medicina57111169)
Supplement: Supplementary file 1 [file medicina-57-01169-s001.zip › Table S4.pdf]

Table S4. Outcomes reported by the included studies (N = 13).

		At risk for MetS		MetS		Reference
		Improved	Total	Improved	Total	
Nutrition	Energy			2	3	[16, 19, 20, 27, 28]
	Alcohol energy			0	1	[19, 20]
	Fat			2	3	[16, 19, 20, 27, 28]
	Saturated fat			1	2	[16, 19, 20]
	PUFA			1	2	[16, 19, 20]
	MUFA			0	1	[19, 20]
	Carbohydrate			1	3	[16, 19, 20, 27, 28]
	Protein			1	3	[16, 19, 20, 27, 28]
	Fibre			2	3	[16, 19, 20, 27, 28]
	Sugar			0	2	[19, 20, 27, 28]
	Cholesterol			0	1	[19, 20]
	Sodium			0	1	[19, 20]
	Glycaemic load			1	2	[19, 20, 27, 28]
	Glycaemic index			1	1	[19, 20]
	Number of meals/day			1	1	[27, 28]
	Slower eating pace			1	1	[27, 28]
	Late-night eating			1	1	[27, 28]
	Breakfast skipping			1	1	[27, 28]
	Reduction in dining out frequency			0	1	[27, 28]
	Reduction in supplement intake			1	1	[27, 28]
	Vegetables	0	1			[29]
	Fruits	1	1			[29]
	Meat	0	1			[29]
	Bread	1	1			[29]
	Dairy	1	1			[29]
	Diet soda	0	1			[29]
	Regular soda	0	1			[29]
	>=5 glasses of water/day	1	1			[29]
	Diet readiness	1	1			[31, 32]
	FFQ score	0	1			[33]
Physical activity	Energy expenditure			0	1	[19, 20]
	Being active			1	1	[27, 28]
	Physical working capacity at 75%			1	1	[19, 20]
	Grip			1	1	[19, 20]
	Mets-equivalent	0	1	1	1	[16, 33]
	Frequency	1	1			[29]
	Duration	1	1			[29]
	Functional reach	1	1			[31, 32]
	Sit to stand	1	1			[31, 32]
	Half sit up	1	1			[31, 32]
	6MWT	0	1			[31, 32]
	Right leg balance	0	1			[31, 32]
	Left leg balance	0	1			[31, 32]
	Vertical leap	0	1			[31, 32]
	Exercise readiness	1	1			[31, 32]
Anthropometry measure	Weight	3	4	4	6	[16-23, 25, 26, 29-32]
	Waist circumference	4	5	7	7	[16-23, 25-33]
	Hip circumference	1	1			[25]
	Waist-hip ratio	1	1	0	1	[19, 20, 25]
	Body mass index	2	4	5	7	[16-23, 25, 26-29, 31-33]
	Body fat mass			1	1	[19, 20]

	Body fat %			0	2	[19, 20, 27, 28]
	Lean			1	1	[19, 20]
	Abdominal fat			1	1	[19, 20]
	Visceral fat area	1	1			[24]
Cardiovascular measures	Systolic blood pressure	4	5	3	6	[16-25, 27, 28, 30-33]
	Diastolic blood pressure	2	4	4	6	[16-25, 27, 28, 30-33]
	MAP			1	1	[19, 20]
	Large AC			0	1	[19, 20]
	Small AC			0	1	[19, 20]
Biomarkers	Fasting blood glucose	2	3	4	6	[16-25, 27, 28, 30]
	Total cholesterol	1	2	2	4	[16-22, 24, 25]
	HDL-cholesterol	1	2	3	6	[16-25, 27, 28]
	LDL-cholesterol	2	2	2	3	[17-22, 24, 25]
	Triglyceride	0	2	2	6	[16-25, 27, 28]
	Fasting insulin	0	1	0	2	[16, 19, 20, 24]
	HOMA-IR	1	1	0	2	[16, 19, 20, 24]
	HOMA-b	1	1			[24]
	c-Reactive protein	1	1	1	2	[16, 21, 22, 24]
	Uric acid			1	1	[16]
	TG: HDL ratio			1	1	[17, 18]
	HbA1c	2	2			[24, 25]
	Hepatic insulin resistance index	1	1			[24]
MetS components	Proportion of MetS	1	1	1	1	[16, 25]
	Central obesity/ elevated waist circumference	1	1	1	1	[16, 25]
	Hypertension /elevated blood pressure	1	1	0	1	[16, 25]
	Hyperglycaemia	0	1	1	1	[16, 25]
	Low HDL-cholesterol	1	1	0	1	[16, 25]
	Hypertriglyceridemia	1	1	1	1	[16, 25]
	Number of MetS components	1	1			[24]
Other outcomes	Longer sleeping duration			1	1	[27, 28]
	Smoking			1	1	[27, 28]
	Smoking readiness	0	1			[31, 32]
	Diabetes readiness	0	1			[31, 32]
	Stress	0	1			[33]
	Literacy / knowledge	2	2	1	1	[26, 30, 33]
	Perception of MetS	0	1			[33]
	Life satisfaction	0	1			[29]
	SF12 score	0	1			[31, 32]
